# Patient-specific neurosurgical phantom: assessment of visual quality, accuracy, and scaling effects

**DOI:** 10.1186/s41205-018-0025-8

**Published:** 2018-03-13

**Authors:** Felipe Wilker Grillo, Victor Hugo Souza, Renan Hiroshi Matsuda, Carlo Rondinoni, Theo Zeferino Pavan, Oswaldo Baffa, Helio Rubens Machado, Antonio Adilton Oliveira Carneiro

**Affiliations:** 1 Department of Physics, Faculty of Philosophy, Science and Letters at Ribeirao Preto, University of Sao Paulo, Av. Bandeirantes, 3900, Monte Alegre, Ribeirão Preto, SP CEP 14040-901 Brazil; 2 Department of Surgery and Anatomy, Faculty of Medicine at Ribeirao Preto, University of Sao Paulo, Ribeirão Preto, Brazil

**Keywords:** Neurosurgery, Education, Medical training, Patient-specific, Neuronavigation, Phantom, 3D printing, Simulator

## Abstract

**Background:**

Training in medical education depends on the availability of standardized materials that can reliably mimic the human anatomy and physiology. One alternative to using cadavers or animal bodies is to employ phantoms or mimicking devices. Styrene-ethylene/butylene-styrene (SEBS) gels are biologically inert and present tunable properties, including mechanical properties that resemble the soft tissue. Therefore, SEBS is an alternative to develop a patient-specific phantom, that provides real visual and morphological experience during simulation-based neurosurgical training.

**Results:**

A 3D model was reconstructed and printed based on patient-specific magnetic resonance images. The fused deposition of polyactic acid (PLA) filament and selective laser sintering of polyamid were used for 3D printing. Silicone and SEBS materials were employed to mimic soft tissues. A neuronavigation protocol was performed on the 3D-printed models scaled to three different sizes, 100%, 50%, and 25% of the original dimensions. A neurosurgery team (17 individuals) evaluated the phantom realism as “very good” and “perfect” in 49% and 31% of the cases, respectively, and rated phantom utility as “very good” and “perfect” in 61% and 32% of the cases, respectively. Models in original size (100%) and scaled to 50% provided a quantitative and realistic visual analysis of the patient’s cortical anatomy without distortion. However, reduction to one quarter of the original size (25%) hindered visualization of surface details and identification of anatomical landmarks.

**Conclusions:**

A patient-specific phantom was developed with anatomically and spatially accurate shapes, that can be used as an alternative for surgical planning. Printed models scaled to sizes that avoided quality loss might save time and reduce medical training costs.

## Background

Medical error can be defined as acts of commission or omission that have the potential to harm or that effectively harm patients [1]. A study has revealed that technical errors, such as problems in equipment use or in the performance of a procedure, cause 27.8% of these events [2, 3]. The image-guided navigation (IGN) systems have been employed to assist surgeons during complex surgical procedures [4] that require extreme manual and visual abilities. IGN allows real-time surgical tool localization through co-registration of the patient’s body and tomographic images, such as computed tomography (CT), magnetic resonance imaging (MRI), and ultrasound imaging (US) [5]. Neuronavigation systems are IGN systems dedicated to help surgeons during neurosurgeries to locate a tool in a three dimensional (3D) space while moving it around or inside the patient’s head [6]. Mastering the use of navigation systems requires extensive training in a controlled environment to allow accurate and precise measurements.

Medical training is another method to minimize medical errors and to make procedures more accurate. Physicians train to develop technical skills and to achieve expertise [7, 8]; that is, physicians practice in order to acquire the ability to reproduce a given task with superior performance [9]. The classical approach to anatomy and surgery teaching and learning is based on the use of human cadavers [10] and animals. However, this strategy is becoming less common due to the high cost and ethical and/or logistic issues involved in collecting cadavers and in maintaining live animals prior to sacrifice. The number of medical training tools has increased significantly in recent years. Learning centers are constantly improving simulation environments to help students and professionals to reach expertise [11–13]. Computational simulation [11, 13–15] and phantoms [16] are alternative tools for training purposes.

Advances in material technology and computer sciences have provided inexpensive alternatives for medical training. Polymers that mimic the elastic and haptic properties of human tissues are examples of such alternatives. The tissue-mimicking materials (TMM) employed in phantoms can reproduce specific physical, chemical, and/or biological properties of the human anatomy. Indeed, TMM can mimic structures that range from homogeneous tissues to complex structures [17, 18]. Several TMM have been reported in the literature [17, 19–22]. Agar/gelatin is a water-based material that is easy to prepare, but it must be protected from bacterial attacks and dehydration. On the other hand, oil-based materials, like styrene-ethylene/butylene-styrene (SEBS) gels, are biologically inert and present tunable properties [20]. The mechanical properties and touch feeling of oil-based materials and biological soft tissues are similar, so the former are adequate for ultrasound applications [21, 23].

The 3D printing in neurosurgery training allows reproduction of the morphology and structural features of specific patient cases by means of segmented CT or MRI images. Even though this technique is versatile, it is not free of limitations, which include the physical properties of the printing material, the cost, and the relation between the manufacturing time and the quality of the printed structure [24]. Involvement of professionals of different medical, technological, and artistic areas is essential to integrate the different devices, software, and protocols that simulate the neurosurgical environment and lead to viable outcomes [25, 26]. Together, these techniques should improve surgical planning, minimize errors, and increase the surgeon’s confidence. Exhaustive training is the best method to achieve expertise and to acquire appropriate skills [7, 13].

The aim of this study was to develop a patient-specific phantom for neurosurgery to reproduce brain cortical morphology, cerebrospinal fluid, meninges and scalp. The model was obtained by 3D printing and SEBS modeling. The quality assessment of the hybrid model was made by inspecting fused images (real and phantom) and by applying a questionnaire to health specialists. Additionally, we assessed the effect of reducing the size of a segmented model in the measurements performed with a neuronavigation system.

## Methods

### Patient-specific phantom

The MRI scan of a three-year-old boy diagnosed with Sturge-Weber Syndrome [27] was acquired using a 3DT1 gradient echo sequence (TR = 4.8 ms; TE = 3.4 ms; acquisition matrix = 129 × 164 × 169 mm; slice thickness = 1 mm; pixel size = 1 × 1 mm) in a scanner Achieva 3 T (Philips, The Netherlands). The MRI scan was segmented to reveal the cortical surface and a representative portion of the patient’s face. A 3D model was reconstructed and exported to stereolithographic file (.STL) with the aid of the InVesalius software (Centro de Tecnologia da Informação Renato Archer, Campinas, Brazil) [28]. By using Blender (Blender Institute, Amsterdam, the Netherlands), the STL model was separated into three pieces: skull with facial information and left and right brain hemispheres [29]. The local ethics committee approved the experimental procedure and the patient’s guardian provided a signed informed consent (CAAE 36460914.4.0000.5440).

The left and right brain hemispheres were printed (PLA fused filament) separately by using a Zmorph 2.0 S (Zmorph LLC, Wroclaw, Poland) printer with a z-layer: 0.1 mm, path width 0.4 mm. The 3D prints were used to make negative molds with a commercial white silicone rubber (Polglass, Ribeirão Preto, Brazil). The molds were filled with molten SEBS gel (Kraton Polymers, Houston, EUA), which was manufactured by mixing the SEBS copolymer with mineral oil at a concentration of 10% *w*/w; as described by Cabrelli et al. [23]. Silicone 3% (*w*/w) (Silaex, São Paulo, Brazil) and aniline were added to improve the mechanical properties and coloring, respectively.

To evaluate the Young’s modulus of the gel, a TA.XT plus Texture Analyzer (Stable Micro Systems, Surrey, UK) equipped with a plate with diameter of 40 mm was used to conduct mechanical tests on a cylindrical sample with diameter of 2.5 cm and thickness of 2 cm.

**Fig. 1:**
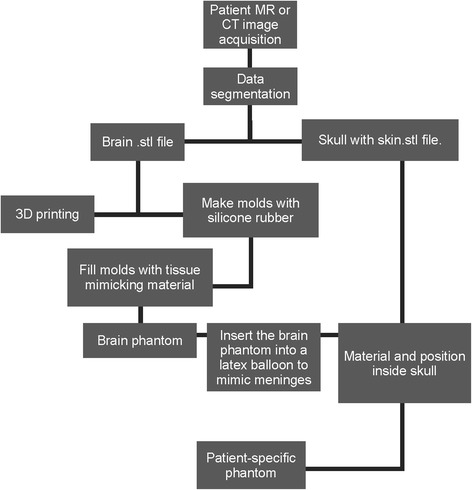
Flowchart illustrating patient-specific phantom production, from patient data acquisition to final specific phantom

The brain phantom was covered with a latex balloon filled with 10% w/w gelatin solution 250 Bloom (Gelita, Eberbach, Germany) to mimic the *dura mater* and the cerebrospinal fluid. This structure was inserted into the PLA 3D-printed patient’s skull and skin. To verify the locations of each structure inside the final model, a CT scan was recorded on a Brilliance Big Bore scanner (Philips Medical Systems, Cleveland, USA) operating at 120 kVp; the slice thickness was 1 mm. The patient-specific phantom CT images were registered and fused with native patient’s T1-weighted anatomical MRI by using fiducial registration in a 3D Slicer (Slicer, Cambridge, USA) [30]. Figure 1 shows the flowchart of all the steps used to build the patient-specific model.

The quality of the resulting anatomy was assessed by visually inspecting the fused images and by comparing the patient-specific phantom images with the real patient’s images. An experienced neurosurgeon (HRM) traced the path for both the skin cut and the craniotomy on the right side of the skull. Next, a window was opened by drilling the indicated path to expose the insula region. Finally, a board of 17 individuals (including 2 neurosurgeons, 6 neurologists, 2 radiologists, 1 pediatric and 6 health professionals) evaluated the quality of the resulting phantom by answering a standard questionnaire. All individuals were instructed to compare with their previous experiences how much the phantom simulate each item, from “nothing” to “perfect”.

### Multiscale models

Three different sizes of 3D-printed models based on a real surgical case were produced to compare the performance during neuronavigation and the cost-benefit ratio regarding time and printing quality. The first model (real-size model, designated B100) was printed in a Paragon device (Rapid Technologies, Middleton St George, UK) by using selective laser sintering; the total printing time was 24 h. Then, a model with half the original size (designated B50) was printed by PLA extrusion in a ZMorph 2.0 S printer (z-layer: 0.1 mm, path width 0.4 mm); the total printing time was approximately 10 h. Finally, a model with one quarter of the original size (designated B25) was printed by PLA extrusion in a Prusa i3 printer (Rep Rap, China) with same presets.

### Neuronavigation protocol

The printed models and neuronavigation were used to assess the effect of scale reduction on the neuroanatomical localization. The navigation was performed by using the open source software InVesalius Navigator [31] installed on a personal computer and connected to the optical tracking device MicronTracker Sx60 (ClaroNav, Toronto, Canada). The images were registered by point-based registration and linearly adjusted to the corresponding scale factor. The fiducial registration error (FRE) was calculated for each procedure to control the navigation quality. The coordinates of nine anatomical landmarks of clinical relevance were digitized during neuronavigation of each model: left and right ear (LE and RE, respectively), LE and nasion (N), RE and N, left and right post-central gyrus (LPG and RPG, respectively), Broadmann Area 44 (BA44), supramarginal gyrus (SMG), and left and right lateral-occipital gyrus (LOG and ROG, respectively). Measurements were performed by three independent raters in three distinct acquisition series each. Raters were physicists with at least four years of experience in neuronavigation and visual analysis of anatomical characteristics of MRI slices.

### Statistical analysis

The Euclidian distances between the six pairs of digitized landmarks were calculated (LE-N, LE-RE, RE-N, LPG-RPG, SMG-BA44, LOG-ROG). Two-way analysis of variance (ANOVA) was used to test whether the measured distance depended on both the scale factor and the target pair of anatomical landmarks. Post hoc Tukey’s test was employed to compare all the groups. Statistical significance was set to 5%.

## Results

### Patient-specific phantom

**Fig. 2:**
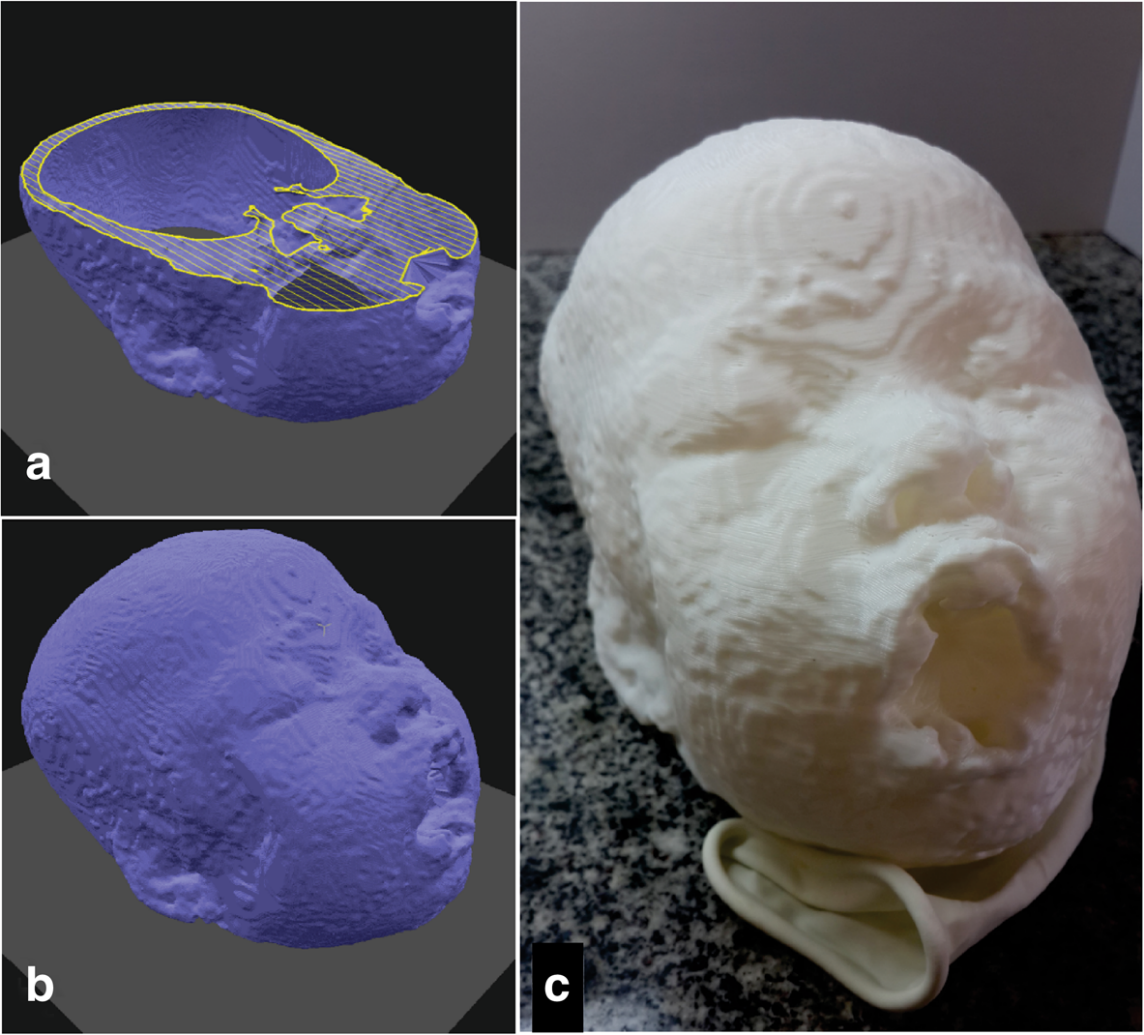
**a** 3D digital planning for printing showing an intermediate layer and **b** the final shape based on patient’s data. **c** Head phantom printed with PLA extrusion

**Fig. 3:**
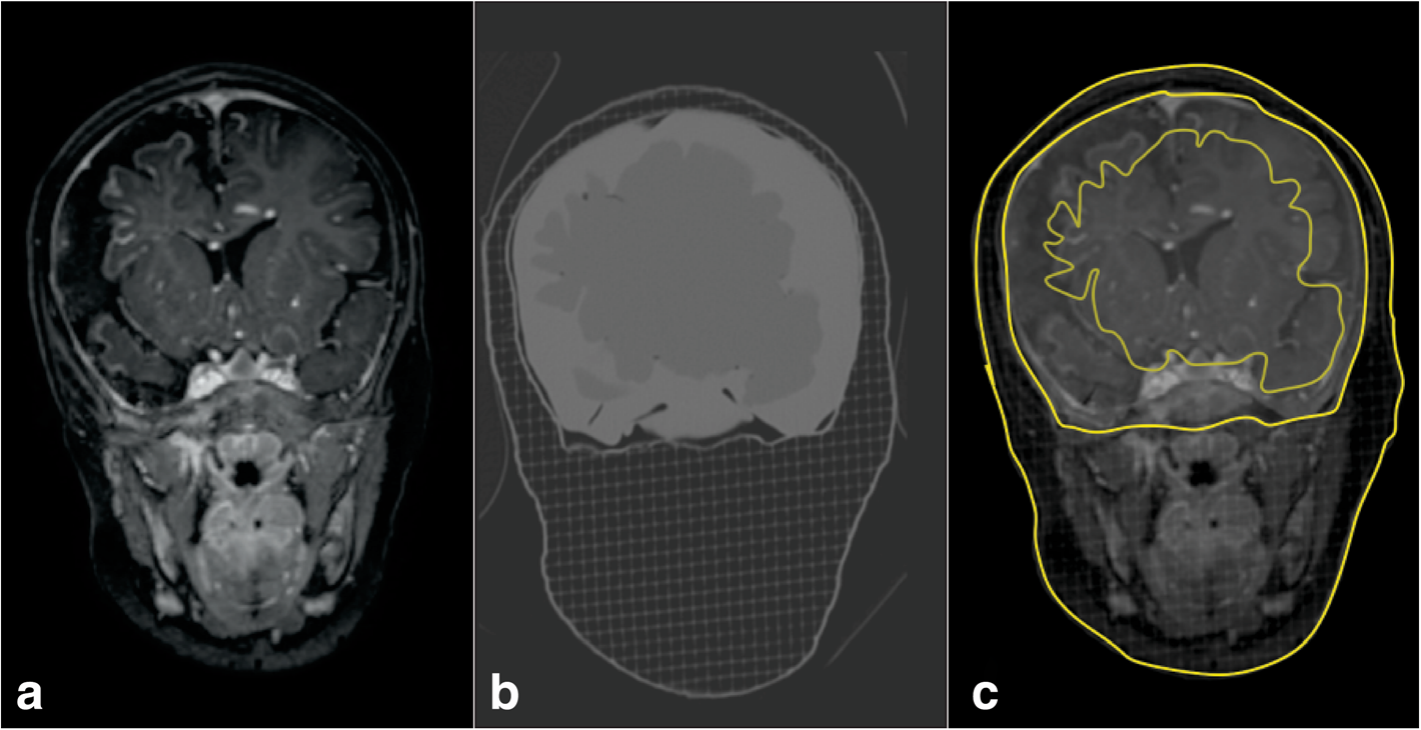
**a** Patient’s MRI. **b** Phantom CT image. **c** Fused images with highlighted phantom CT image contour

The molten SEBS gel used to to obtain the morphological brain phantom has Young’s modulus 32 ± 1 kPa. Figure 2a shows an intermediate layer of the printing plan, Fig. 2b depicts the final printing plan and Fig. 2c illustrates the printed head. We took the CT images of the final model and registered them with the patient’s MRI. Figure 3 illustrates the fiducial registration of the patient-specific phantom CT images and the patient’s MRI.

**Fig. 4:**
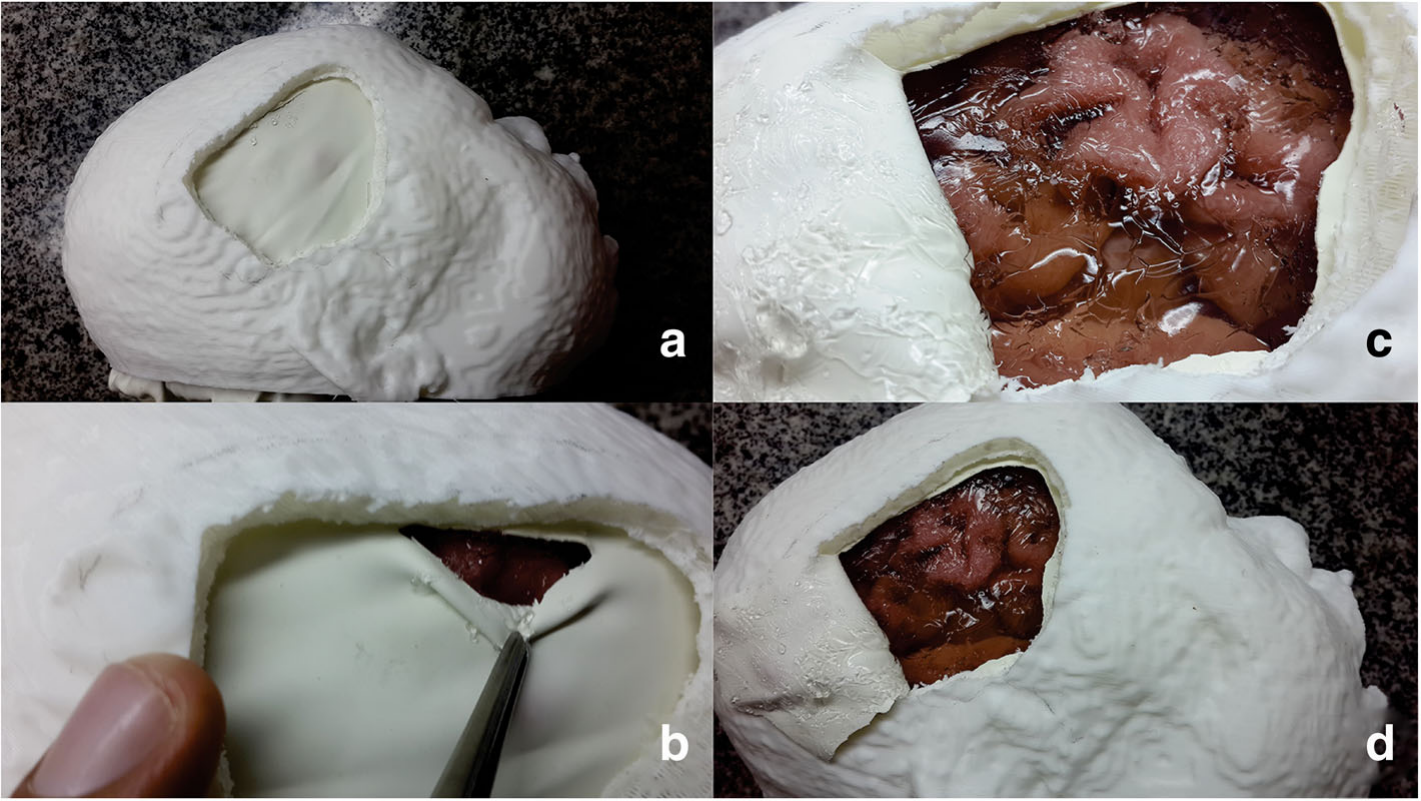
Visual aspect of the mounted patient-specific model. **a** Lateral view after craniotomy; **b** initial cut of the rubber- mimicked meninges; **c** exposed surface of the brain tissue and cerebrospinal fluid mimicking; **d** overview of the resulting patient-specific realistic phantom

Figure 4a shows the model after the craniotomy performed according to the neurosurgeon’s indication. In addition, we can see the structure mimicking the *dura mater* (Fig. 4b), the insula exposure (Fig. 4c) and the patient-specific phantom after the craniotomy simulation (Fig. 4d).

Table 1 lists the questionnaire results. The neurosurgeons team evaluated the phantom realism as “very good” and “perfect” in 49% and 31% of the cases, respectively. The neurosurgeons rated the phantom as “very good” and “perfect” in 61% and 32% of the cases, respectively. It is relevant to observe that, the grade “perfect” is an individual perception considering the purpose of the models. Therefore, it might not reflect that the model has the same properties as cadaveric models or real human tissues.

**Table 1:** Results of patient specific phantom assessment

**Assessment of phantom realism**					
	Nothing	Not Good	Good	Very Good	Perfect
Phantom general aspect (anatomical structures: proportions and locations)	0%	0%	24%	47%	29%
Haptic response	0%	0%	18%	59%	24%
Size of the brain internal structures	0%	0%	18%	53%	29%
Brain tissue appearance	0%	0%	18%	53%	29%
Relationship between internal anatomical structures used as reference for access	0%	0%	24%	35%	41%
Realism average	0%	0%	20%	49%	31%
**Assessment of phantom educational potential (utility)**					
	Nothing	Not Good	Good	Very Good	Perfect
To acquire basic skills necessary for surgery	0%	0%	6%	65%	29%
To acquire depth sensation (insertion / extraction) through the bony window	0%	6%	6%	59%	29%
To learn how to orient yourself during the surgical procedure	0%	0%	6%	59%	35%
To learn procedures	0%	0%	6%	65%	29%
To learn how to position drains and other equipment	0%	0%	6%	59%	35%
Utility Average	0%	1%	6%	61%	32%
Overall Average	0%	1%	13%	55%	31%

### Multiscale models

**Fig. 5:**
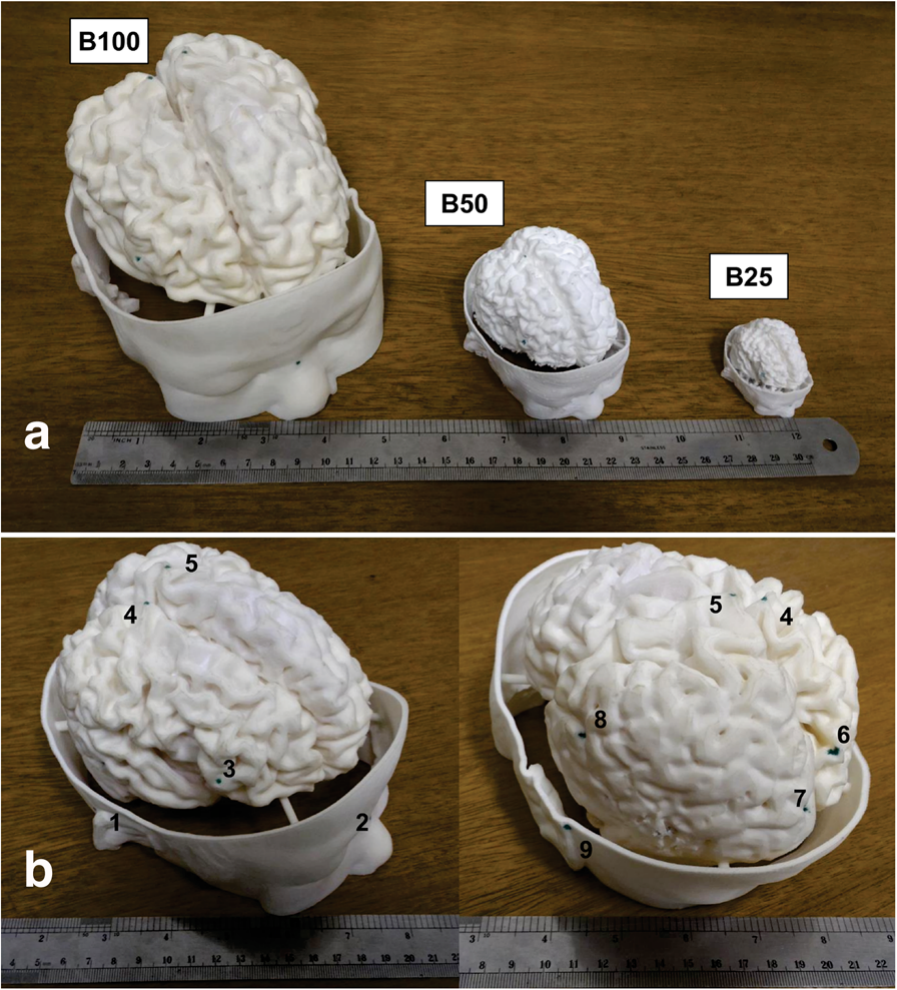
Scaled models used during neuronavigation. **a** Original size (B100), half-sized (B50) and quarter-sized (B25) models. **b** Frontal and back view of anatomical landmarks used for navigation and measurements: 1. right ear, 2. nasion, 3. right inferior frontal cortex, 4. right parietal cortex, 5. left parietal cortex, 6. right occipital cortex, 7. left occipital cortex, 8. left inferior frontal cortex, 9. left ear

Figure 5 shows all the models we used during the neuronavigation protocol. Inspection of the model B50, printed with PLA extrusion, and of the model B100, manufactured by selective laser sintering, provided a quantitative and realistic visual analysis of the patient’s cortical anatomy without any distortion due to scale reduction. However, the model B25, which corresponded to one quarter of the original size, had reduced surface details, which hindered identification of the anatomical landmarks. Reduced model size drastically decreased the printing time, which was 3, 10, and 22 h for B25, B50, and B100, respectively.

The estimated FRE for neuronavigation (mean ± standard deviation) was 2.5 ± 0.3, 1.7 ± 0.2, and 1.5 ± 0.3 mm for B25, B50, and B100, respectively. Two-way ANOVA revealed that the calculated distance depended on both the scale factor (F_2.144_ = 40.56; *p* < 0.001) and the pair of anatomical landmarks (F_5.144_ = 55,951.70; *p* < 0.001). Also, we found a significant interaction between the pair of fiducial coordinates and the scale factor (F_10.144_ = 11.78; *p* < 0.001). Multiple comparison tests revealed that the LE-RE distance was greater in B25 as compared to B50 and B100 (95% confidence interval 125.8–129.6, *p* < 0.001). The difference between the LE-RE distances in B25 and B50 and in B25 and B100 was 2.6 and 2.7 mm, respectively. The RE-N distance was greater for B25 than for B50 and B100 (95% confidence interval 124.0–127.8, *p* < 0.001); the mean difference between the RE-N distances in B25 and B50 and in B25 and B100 was 5.8 and 5.1 mm, respectively. B25, B50, and B100 did not differ significantly in terms of the remaining pairs of anatomical landmarks. In general, the measured distances tended to display higher deviations in the smallest model as compared to B50 and B100.

## Discussion

In an attempt to minimize medical errors, the simulation of medical procedures has become a common approach in medical training. In this scenario, simulating the specific characteristics of a patient constitutes a challenge [9]. The use of negative molds is a well-known method to reproduce morphology [32, 33]. However, it is impossible to copy internal structures or organs for in-vivo studies of human cases. The use of 3D printers enables the development of realistic phantoms with anatomically and spatially accurate shapes [34]. Previous studies have reported the use of 3D printing to reproduce the patient’s anatomy for surgical and treatment planning and to facilitate understanding of the normal and pathologic anatomy of individual patients in different situations, such as aneurysm [35, 36]. Weinstock et al. used 3D printing and silicone molding and relied on the aid of Hollywood special effects technicians, who finished the model with makeup, to improve realism [37]. Therefore, the combination of 3D printing and molding technique helps to accelerate object manufacture [37]. Compared to 3D printing, in this study the silicone molds enabled faster production of multiple copies with the same details and even allowed the preparation of materials that are not yet available for 3D printing. We also verified that the soft tissue-mimicking material SEBS bore a striking resemblance to the patient’s brain cortex anatomy and enabled haptic feedback for surgical procedures. Further development should allow mimicking of internal brain structures and dynamic functions, such as blood circulation.

External details of the PLA printed face provided anatomical landmarks that may help surgeons to plan the surgery, thereby improving realistic training. We planned the craniotomy on the basis of the traditional pterional craniotomy approach. Figures 4b and c show that the virtual planning and the final phantom were very similar. To improve the total time, we used a printing layer of 0.1 mm. If necessary, layers of 0.03 mm might be used to achieve a better level of details. Virtual printing planning is not always identical to the final printed piece [38]. Printing errors may cause deformations, overlaps, or missing areas as compared to the planning file [24]. Due to these errors, the layer height and other parameters, such as infill ratio, printing speed, and support settings should be considered for each case, and the printed object must be analyzed before it is used. In this study, another important observation concerned the dimensions of the object to be printed; the patient-specific model presented herein almost reached the limit of the 3D printer (Zmorph - printing area 300 × 235 × 165 mm). Therefore, manufacturing models of older or bigger patients would demand that pieces subdivided for printing and then used to mount the final phantom.

Fusion of the phantom CT images and of the native T1-weighted MRI scans revealed a brain shift in the patient-specific phantom as compared to the real patient’s data. Figure 3b highlights the difference between the patient’s MRI scan (Fig. 3a) and the phantom CT image (Fig. 3c). Future development of the procedure presented here should improve the methods for brain positioning inside the skull. Fixed guiding points could improve brain mounting by diminishing the shift in relation to the original anatomy. Another possibility would be to use neuronavigation to refine orientation during model construction.

Patient-specific phantom assessment revealed a didactic potential for medical training, mainly for unusual diagnosis. The insertion of individual characteristics during training for a specific surgery might minimize serious errors [1] like operating on the wrong side [39] or on the wrong patient. Moreover, phantoms allow for exhaustive training by repetition of a specific procedure step anywhere and anytime. Not only surgeons but the entire medical team can practice with patient-specific phantoms, thereby increasing the total training time and respective individual learning curves [40].

Multiscale models have been developed and tested for neurosurgery training with neuronavigation. The InVesalius Navigator software enables real-time localization and digitization of anatomical structures with fiducial registration errors within the limits recommended by the specialized literature for clinical practice, i.e. below 3 mm [41]. Neuronavigation on B50 and B100 provided more accurate and precise distance measurements as compared to neuronavigation on B25. The smallest model showed higher deviations for the measurements, specifically for the LE-RE and the RE-N distances. Both pairs of anatomical landmarks had the right ear as one of the references, which suggested that a possible systematic deviation was associated with the acquisition of the coordinates in B25. Indeed, we expected that B25 would provide lower-quality results as compared to B50 and B100 because the small scale approximated all the measurements to the uncertainty levels. Furthermore, none of the remaining references showed any difference associated with distance measurements across scale factors. A possible explanation is that visually localizing the same reference points on the smallest model is more difficult compared to the larger ones. We should emphasize that B50 had enough accuracy for simulation purposes. Reducing the scale of printed models without quality loss might help to reduce the protocol duration and the costs in future iterations. Thus, neuronavigation with B50 might be a suitable alternative to the real size model because it provided similar visual detailing.

It is important to note that, direct comparison between production time and measurement might be limited because the three scaled models were produced using different devices and materials. Nevertheless, it is expected that production time is shorter for smaller models, and different materials might have a small impact in measurements during neuronavigation as the resolution from the printing devices are significantly greater than from the measurement device.

## Conclusion

A patient-specific (Sturge Weber case) phantom was successfully created by using 3D printing techniques and molds that included a soft tissue mimicking material for the brain structure, meninx and skull. Our study revealed that 3D printing and SEBS are promising tools to develop a patient-specific phantom as a teaching tool, providing tissue mimicking material and reliable anatomical morphology. Moreover, the molding technique enabled the performance of fast copies for a single case, which confirmed the didactic potential of the presented models. The use of multiscale models is a successful alternative to improve time and to simulate the general steps of surgical procedures.

## Abbreviations

3D: Three dimensional
BA44: Broadmann Area 44
CT: Computed tomography
FRE: Fiducial registration error
IGN: Image-guided navigation
LE: Left ear
LOG: Left lateral-occipital gyrus
LPG: Left post-central gyrus
MRI: Magnetic resonance imaging
N: Nasion
PLA: Polyactic acid
RE: Right ear
ROG: Right lateral-occipital gyrus
RPG: Right post-central gyrus
SEBS: Styrene-ethylene/butylene-styrene
SMG: Supramarginal gyrus
STL: Stereolithographic file
US: Ultrasound imaging
